# Risk and impact of herpes zoster among COPD patients: a population-based study, 2009–2014

**DOI:** 10.1186/s12879-018-3121-x

**Published:** 2018-05-03

**Authors:** Cintia Muñoz-Quiles, Mónica López-Lacort, Javier Díez-Domingo

**Affiliations:** 1 Vaccine Research. Fundación para el Fomento de la Investigación Sanitaria y Biomédica de la Comunitat Valenciana, FISABIO-Public Health, Valencia, Spain; 2 Universidad Católica de Valencia San Vicente Mártir, Carrer de Quevedo, 2, 46001 València, Spain; 3 Vaccine Research Area, FISABIO-Public Health, Avda. Cataluña, 21, 46020 Valencia, Spain

**Keywords:** Herpes zoster (HZ), Chronic obstructive pulmonary disease (COPD), Epidemiology, Population-based study, Vaccine

## Abstract

**Background:**

The objective of this study was to assess the incidence of Herpes Zoster (HZ) among patients with chronic obstructive pulmonary disease (COPD) and the impact of HZ on the underlying COPD.

**Methods:**

A retrospective cohort of all subjects older than 49 years was followed up between 2009 and 2014 using population and health databases of Valencia Region (Spain). HZ and COPD were identified using ICD-9 codes, differentiating COPD patients with inhaled corticosteroids prescriptions (COPD-ICS). The incidence of HZ was compared among 3 groups [non-COPD, COPD and COPD-ICS populations] and use of healthcare resource due to HZ for 6 months following HZ diagnosis through different statistical generalized linear models (GLM). We also compared resources consumption due to COPD before and after HZ.

**Results:**

The cohort consisted of 2,289,485 subjects, including 161,317 COPD patients of which 29,708 were COPD-ICS. HZ incidence rates were 11 (95% confidence interval [CI]: 10.7–11.4) and 13 (95% CI: 12.3–13.8) cases/1000 persons-year for COPD and COPD-ICS populations respectively. Incidence increased with age in all groups. The risk of HZ rose by 45 and 61% among COPD and COPD-ICS patients respectively compared to non-COPD (95% credible intervals [CrI]: 1.41–1.5 and 1.52–1.71 respectively). COPD patients consumed more resources due to their HZ than non-COPD. There was no statistically significant impact of the HZ on the resources consumed due to COPD during the 6 months post-HZ compared to the 6 months pre-HZ.

**Conclusions:**

The presence of COPD increases the risk, severity and impact of zoster episodes.

**Electronic supplementary material:**

The online version of this article (10.1186/s12879-018-3121-x) contains supplementary material, which is available to authorized users.

## Background

Herpes Zoster (HZ) is a severe disease resulting from the reactivation of Varicella Zoster Virus (VZV), which remains latent in the nervous system after primary infection (Varicella) [1]. This reactivation seems to be a result of a waning of VZV-specific cell-mediated immunity [2], as occurs with ageing or in individuals with immunosuppressive conditions. HZ can result in chronic pain which is its most common complication (post-herpetic neuralgia, PHN) [3]. Many patients with PHN develop severe physical, occupational and social disabilities because of enduring pain. HZ and PHN reduce life quality, and increase healthcare costs [4].

Incidence of HZ increases strongly with age, being higher after the age of 50 and affecting up to 50% of people who live to 85 years [5, 6]. Beyond age, concomitant diseases increase the risk, the severity and the impact of HZ in advanced age population. Diabetes mellitus, chronic obstructive pulmonary disease (COPD) or cardiovascular diseases have been identified as risk factors for HZ and PHN [7–10]. The prevalence and the impact of chronic diseases have been increasing constantly the last decades [11, 12]. Maintaining the affected population healthy constitutes a priority [13, 14], avoiding exacerbations provoked by infectious diseases, which might render the affected individual dependent on their families or the healthcare system.

Vaccinations represent important preventive tools against infections. A live-attenuated vaccine for the prevention of HZ (Zostavax, Sanofi Pasteur MSD) was approved in 2006 and has been since licensed in many countries for adults older than 50 years of age [15, 16]. Its effectiveness has been assessed in several studies, which showed similar results in healthy subjects and individuals with chronic diseases [17–19]. Currently in Spain, there are efforts to introduce recommendations for HZ vaccination. Given the relatively high cost of the zoster vaccine the identification of populations at increased risk for HZ constitutes an important research question.

COPD is the fourth cause of death worldwide, projected to be the third by 2020, and ranks fifth in terms of disease burden [12]. COPD patients have higher risk of being affected by invasive diseases and have frequently co-morbidities making COPD management complicated. There is evidence that autoimmunity plays a role in the pathogenesis of COPD [20]. The increased numbers of T-cells and B-cells in the lungs of COPD patients, the detection of auto-antibodies in their serum and the findings in animal models [20–22] support this hypothesis. This immune system impairment could be associated with an increased HZ risk in COPD patients.

Few studies have addressed the risk of developing HZ in COPD patients, but none the impact of COPD on the severity, complications of HZ and healthcare expenditure. Moreover, their results regarding the relationship between HZ and the usage of inhaled corticosteroids (ICS) in severe COPD are controversial [7, 23–25]. In this population-based study we considered the COPD impact on the risk and severity of HZ, estimating the HZ incidence in these patients and the healthcare resources utilization attributed to the HZ. We also assessed the HZ impact on the COPD patients by looking at the ensuing exacerbation of the disease, measured in terms of increased outpatient visits, hospitalizations and medication prescriptions after the HZ episode. These relationships were evaluated considering COPD patients with and without ICS treatment (COPD-ICS).

## Methods

This is a population-based study to assess the impact of underlying COPD on the risk of suffering HZ, the associated healthcare resources use, and the HZ impact on COPD patients using databases of the Valencia Region. The methods used in this study were similar to those used in a previous work from our group [10], briefly:

### Setting and study population

In 2015 the Valencia Region of Spain had 4,980,689 inhabitants [26] registered. Over 98% were insured by the Regional Health System (RHS) [27], which consists of 24 Health Departments [10]. All visits to primary care and hospital admissions are recorded in relevant clinical databases.

Around 37% of the population in the Valencia Region is ≥50 years old. The cohort of the study included all subjects’ aged 50 years or older, insured by the RHS and living in the Valencia Region between 1st of January 2009 and 31st of December 2014. We excluded patients with immunosuppressive conditions [10, 28].

### Data source: Electronic databases

Primary care electronic medical records (SIA) and all medical visits with ICD-9-CM recorded diagnoses are registered [29]. The minimum basic data set (MBDS) was used for hospitalization, which collects diagnosis and procedures as an assessment of medical activity also ICD-9-CM coded. For medication information we used the Care Provision Management (GAIA) created by the Department of Health, which contains all the registers regarding prescription and dispensation. The unique personal identification number (SIP) allows to link data from all these databases [6, 10].

### Case definitions

The first appearance of an ICD-9-CM code related to HZ (053.*) in SIA or MBDS was considered an incident case of HZ [10]. When a HZ code appeared 6 months after a previous HZ registry, it was considered a recurrent HZ case [6, 10].

COPD patients were defined when a diagnostic ICD-9-CM code for COPD (491, 492 or 496) was recorded in SIA or in MBDS. A COPD case was considered severe, when an inhaled corticosteroids prescription (COPD-ICS) was issued.

### Variables

Gender, age, health department, nationality, social exclusion risk and rural/urban residence were recorded for each individual [10]. Diabetes and heart failure (HF) were considered also as co-variables and patients were identified when a diagnostic ICD-9 code for HF (428–428.9) or for diabetes (250.*) was detected and when a prescription or dispensation for insulin and/or oral anti-diabetic drugs was recorded in GAIA.

### Statistical analysis

The statistical analysis was described previously [10]:

Incidence rates of HZ and 95% CI were estimated by sex, age and year by the Poisson exact method. We calculated and compared the HZ incidence among non-COPD, COPD and COPD-ICS populations and the healthcare resource use caused by HZ during 6 months following the HZ diagnosis. Recurrence rates of HZ were also estimated [10].

The risk of suffering HZ in COPD patients was calculated using Bayesian mixed Poisson regression, adjusting by year, gender, health department (as a random effect), other co-morbidities (diabetes and HF) and group variable as a random effect to resolve the over-dispersion issue [10].

To compare the use of healthcare resources between the groups we focused on the number and length of hospitalizations and number of outpatient visits with a HZ code, prescriptions for HZ and number and duration of sick leave due to HZ during the six months after the HZ diagnosis (See Additional file 1 for ATC codes, drug type and drug name of the medication registered for HZ and PHN). In order to compare the COPD and the non-COPD groups we performed different GLM depending on the outcome variable (The GLM details are showed in Additional file 2).

The impact of the HZ on the COPD was evaluated in COPD subjects during the six months pre- and post-HZ. We compared the healthcare resources utilization between both periods. In order to compare the pre- and post-HZ periods different GLM were implemented (For GLM details see Additional file 2) [10].

### Ethical considerations

The study protocol was approved by the Ethics Committee of Dirección General de Salud Pública / Centro Superior de Investigación en Salud Pública.

## Results

The final cohort included 2,289,485 Valencia Region residents, between 1st of January 2009 and 31st of December 2014. They were older than 50 years of age. This cohort included 161,317 COPD patients of whom 29,708 were COPD-ICS (18.4%). 67% of COPD and 57% of COPD-ICS were male. Table 1 shows their demographic characteristics.

**Table 1:** Demographic characteristics for population ≥ 50 years old in the Valencia Region from 2009 to 2014 (*n* = 2,289,485)

	Total Cohort	COPD patients (without ICS)	COPD-ICS patients
Age (years)	N (%)	N (%)	N (%)
50–59	1,027,417	26,724	4041
60–69	881,947	47,698	9181
70–79	699,885	59,753	12,928
≥80	414,036	47,289	11,366
Gender			
Male	1, 061,732 (46%)	96,902(67%)	16,913 (57%)
Female	1, 227,753 (54%)	47,628 (33%)	12,795 (43%)
Nationality	^a^ *N* = 1,774,750	^a^* N* = 110,366	^a^* N* = 23,425
Spanish	1, 586,043 (89%)	103,658 (94%)	22,084 (94%)
Other	188,707 (11%)	6708 (6%)	1341 (6%)
Urban status	^a^* N* = 2,261,243	^a^* N* = 144,179	^a^* N* = 29,698
Urban	2, 191,837 (97%)	139,515 (97%)	28,619 (96%)
Rural	69,406 (3%)	4664 (3%)	1079 (4%)
Social Exclusion	^a^* N* = 1,739,725	^a^* N* = 109,603	^a^* N* = 23,301
Risk	202,365 (12%)	9521 (9%)	1754 (8%)
No risk	1, 537,360 (88%)	100,082 (91%)	21,547 (92%)
Comorbidities			
Diabetes mellitus	397,414 (17%)^b^	43,553 (30%) ^b^	9415 (32%) ^b^
Heart Failure	103,240 (5%) ^b^	25,977 (18%) ^b^	7101 (24%) ^b^
Total (%)	2,289,485	144,530 (6%) ^c^	29,708 (1.3%) ^c^

^a^Number of subjects with available information for each category

^b^Percentages calculated considering total of each column as 100%

^c^Percentages calculated considering total cohort (2289485) as 100%

### Underlying COPD impact on HZ

There were 69,438 HZ incident cases, corresponding to an incidence rate (IR) of 7.2 cases/1000 persons-year (95% CI: 7.2–7.3). Of them, 4629 HZ cases (6.7%) were registered in the COPD non-ICS population (IR 11 cases/1000 persons-year; 95% CI: 10.7–11.4) and 1164 (1.7%) in COPD-ICS (IR 13 cases/1000 persons-year; 95% CI: 12.3–13.8). HZ IR increased with age in all 3 groups and was higher in women (Table 2). HZ recurrence rates were 4.2, 5.1 and 5.2 per 100 persons-year for non-COPD (95% CI: 4.1–4.3), COPD (95% CI: 4.7–5.1) and COPD-ICS (95% CI: 4.8–5.5) respectively.

**Table 2:** Incidence rates of HZ (per 1000 persons - per year) by age groups and sex in the Valencia region in 2009–2014

	Non-COPD			COPD (without ICS)			COPD-ICS		
Age (years)	Cases	IR	95% CI	Cases	IR	95% CI	Cases	IR	95% CI
50–59	17,907	5.3	5.3–5.4	452	7.4	6.7–8.1	106	10.6	8.7–12.8
60–69	19,638	7.3	7.2–7.4	1155	10.3	9.7–10.9	257	11.6	10.2–13.1
70–79	16,149	8.3	8.2–8.4	1667	12.0	11.4–12.6	431	13.8	12.5–15.2
≥ 80	9951	8.6	8.5–8.8	1355	12.8	12.1–13.5	370	14.2	12.8–15.7
≥ 50	63,645	7.0	6.9–7.0	4629	11.1	10.7–11.4	1164	13.0	12.3–13.8
Gender									
Male	23,057	5.6	5.5–5.7	2932	10.3	10.0–10.7	581	11.7	10.8–12.7
Female	40,588	8.1	8.0–8.2	1697	12.6	12.0–13.2	583	14.6	13.5–15.9
	Cases	ReR	95% CI	Cases	ReR	95% CI	Cases	ReR	95% CI
≥ 50	6396	4.2	4.1–4.3	612	5.1	4.7–5.1	171	5.2	4.8–5.5

*CI* Confidence Interval, *ReR* Recurrence rate per 100 persons (with a previous incident HZ) - per year

*IR* Incidence Rate

The adjusted risk of HZ increased by 45% among COPD patients and by 61% among COPD-ICS with respect to non-COPD; relative risk (RR) 1.45, 95% CrI: 1.41–1.5; RR 1.61, 95% CrI: 1.52–1.7 respectively (Table 3). The HZ risk was 36% higher in women; it increased with age and in patients with diabetes (20%) and heart failure (25%).

**Table 3:** HZ adjusted relative risk for COPD and COPD-ICS patients in relation with Non-COPD

		Relative Risk (95% CrI)
COPD	NO COPD	1
	COPD	1.45 (1.41–1.5)
	COPD-ICS	1.61 (1.52–1.71)
Sex	Male	1
	Female	1.36 (1.33–1.38)
Age	50–59	1
(Years)	60–69	1.38 (1.34–1.41)
	70–79	1.53 (1.49–1.57)
	80_+	1.49 (1.45–1.54)
Comorbidity		
Diabetes	No Diabetes	1
	Diabetes	1.2 (1.17–1.22)
HF	No HF	1
	HF	1.25 (1.2–1.3)

Relative risk estimates and 95% credible intervals (95% CrI) for the association between HZ incidence and COPD, controlling for sex, age, comorbidities (Diabetes and HF), health department (as a random effect) and calendar year, using Bayesian Poisson regression

COPD patients with HZ utilized more healthcare resources than non-COPD patients (Table 4): they had 5% more outpatient visits, were prescribed 25% more antivirals and had higher risk for hospitalization with a HZ code at any diagnostic position (Odds ratio [OR] 2.7, 95% CI: 2.2–3.2). COPD-ICS subjects had 7% more outpatient visits, 45% more antiviral prescriptions and higher risk for hospitalization with a HZ code at any diagnostic position (OR 2.6, 95% CI: 1.8–3.6). Length of hospital stay and duration of sick leave were not significantly higher in COPD than in non-COPD patients.

**Table 4:** HZ-Health care resources consumption by global COPD (with and without ICS) and COPD-ICS patients in relation with Non-COPD

	COPD	COPD-ICS
Outpatient visits for HZ	RR (95% CI)	
	1.05 (1.03–1.08)	1.07 (1.03–1.1)
Medication for HZ	RR (95% CI)	
	1.25 (1.19–1.31)	1.45 (1.32–1.59)
Hospitalizations^a^	OR (95% CI)	
	2.66 (2.17–3.24)	2.59 (1.80–3.62)
Length of hospital stay	Mean ratio (95% CI)	
	1.21 (0.97–1.52)	1.26 (0.84–1.87)
Sick leave	Mean ratio (95% CI)	
	1.06 (0.62–1.82)	1.06 (0.34–3.29)

*CI* Confidence interval, *RR* Relative risk, *OR* Odds ratio

^a^Hospitalizations with a HZ CIE-9 code in any diagnostic position

### HZ impact on underlying COPD

Outpatient visits (RR 1.02; 95% CI: 0.96–1.08) and number (OR 1.13; 95% CI: 0.96–1.33) and length (OR 1.19; 95% CI: 1–1.42) of hospitalizations for COPD were higher after an HZ episode (Table 5), although the increase was not statistically significant. These results suggest that HZ may have a small and non-significant impact on the underlying COPD.

**Table 5:** COPD-Health care resources consumption during 6 months pre and 6 months post-HZ for COPD cohort

	PRE-HZ	POST-HZ
Outpatient visits for COPD	RR (95% CI)	
	1	1.02 (0.96–1.08)
Hospitalizations ^a^	OR (95% CI)	
	1	1.13 (0.96–1.33)
Length of hospital stay	Mean ratio (95% CI)	
	1	1.19 (1–1.42)
Medication (ICS)	RR (95% CI)	
	1	0.98 (0.76–1.25)

^a^Hospitalizations with a COPD CIE-9 code in any diagnostic position

## Discussion

Patients with COPD are more susceptible to infections than those without [21]. This population-based study demonstrated that COPD increases the risk and severity of HZ episodes and indicated that HZ may contribute to COPD exacerbations resulting to an increase in healthcare resources consumption.

In our study, the IR of HZ was higher in COPD patients older than 50 years and was almost double in COPD-ICS compared to those without COPD. Poisson regression analysis showed that the adjusted HZ RR was 1.5 and 1.6 times higher in COPD and in COPD-ICS patients respectively than in non-COPD. These data are consistent with a previous Spanish study [8], although their adjusted IR ratio was slightly lower than ours, probably due to methodological differences. For instance, they used databases from primary care, whereas we collected data from both, primary care and hospitalizations. Also they included population as young as 18 years old.

Various studies have identified COPD as risk factor for HZ [7, 23, 24, 30] because of systemic immune disturbances in COPD. Recently it was demonstrated that imbalances between subsets of CD8+ peripheral blood T-cells contribute to the pathogenesis of the immune response dysfunction in COPD [31, 32]. Cellular immunity is crucial to maintain VZV in a latent state and impede its reactivation [2]. These results suggest that COPD patients might have an impaired cell-mediated immunity. We also found higher HZ recurrence rate in COPD than in non-COPD subjects, which seems to support this hypothesis.

Yang et al. also found higher HZ risk in COPD patients using ICS [24], the risk being higher in patients using oral corticosteroids. However, other studies have not found this association between HZ and ICS [23, 25]. Ernst et al. pointed to methodological problems, such as insufficient sample size and outcome or exposure misclassification. Two reasons could be responsible for this kind of association. Firstly, ICS possibly affects cellular immunity in COPD-ICS patients, which could increase the HZ risk. Secondly and equally likely, patients using ICS might have a more severe COPD state, making them more susceptible to develop HZ.

To our knowledge, this is the first time that healthcare resources consumption due to HZ is compared between non-COPD and COPD patients. COPD patients attended more frequently outpatient clinics due to HZ than non-COPD and received more antiviral medications. This amount increased in COPD-ICS patients. These data indicate that HZ episodes were more severe in COPD patients and even more in COPD-ICS.

Our results support a recent prospective cohort study which demonstrates that chronic conditions as diabetes, cardiovascular and respiratory diseases increase zoster-related pain and impact the quality of life in immunocompetent zoster patients aged 50 years or older [33]. However, this study was not powered to detect differences between the groups of patients with and without underlying conditions.

Our study also showed a non-statistically significant tendency to more frequent visits to primary care clinics, higher number of hospitalizations and longer hospital stays due to COPD after the HZ episode. Although inconclusive, these results could indicate a negative relationship between HZ and COPD.

Considering the presented results, HZ/PHN vaccination could be helpful when handling these patients. It reinforces the cell-mediated immunity against the latent VZV, preventing thus its reactivation and the related complications. Currently, the vaccine is used to prevent HZ/PHN in USA and various European countries in patients older than 50 years and in those with COPD, diabetes or cardiovascular disease. A recent Spanish study [34] revealed that a vaccination strategy including at least 30% of adults older than 50 years would be cost effective and would increase the benefits of the vaccination program for adults older than 65 years.

Our study has some limitations. As in other studies using routinely collected data, potential codification errors may occur because the data were not created for research purposes. In our case, qualified personnel codify MBDS with almost no mistakes and positive predictive value of those databases’ diagnostic codes resulted high in previous evaluations [6, 10, 35]. Our databases did not allow us to identify the severity of COPD; therefore, we selected patients using ICS considering them to suffer with more severe COPD than patients without ICS treatment. Information about treatment with oral corticosteroids was not available from our database as they are more likely to be administered at hospitals; our pharmacy database collects prescriptions and dispensations from the outpatient services.

## Conclusion

These results reflect that COPD patients have higher risk of suffering HZ and consume more healthcare resources compared to non-COPD patients, which could be the result of a more severe HZ. COPD patients should be considered as a significant risk group for HZ. Our study could represent the first step to assess the impact of a future implementation of the HZ vaccine in COPD population.

## Additional files

Additional file 1: Medication registered for HZ and PHN (ATC codes). Table listing the ATC codes, drug type and drug name of the medication registered for HZ and PHN. (DOCX 14 kb)

Additional file 2: Generalized linear models. Table including the different statistical GLM utilized to compare the COPD and the non-COPD populations depending on the outcome variable. (DOCX 15 kb)

## Abbreviations

CI: Confidence interval
COPD: Chronic obstructive pulmonary disease
COPD-ICS: Chronic obstructive pulmonary disease with inhaled corticosteroids
Crl: Credible interval
GAIA: Care provision management (Sistema de información farmacéutica)
GLM: Generalized linear models
HF: Heart failure
HZ: Herpes zoster
ICD-9-CM: International classification of diseases 9th. Clinical modification
IR: Incidence rate
MBDS: Minimum Basic Data Set (Conjunto mínimo básico de datos)
OR: Odds ratio
PHN: Post-herpetic neuralgia
ReR: Recurrence rate
RHS: Regional Health System
RR: Relative risk
SIA: Ambulatory information system (Sistema de información ambulatoria)
SIP: Personal identification system
VZV: Varicella zoster virus
