# Supplementary material for: Risk and impact of herpes zoster among COPD patients: a population-based study, 2009–2014
Source: BMC Infect Dis. 2018 May 3;18:203. doi: 10.1186/s12879-018-3121-x (PMC5934818; doi:10.1186/s12879-018-3121-x)
Supplement: Supplementary file 1 — Medication registered for HZ and PHN (ATC codes). Table listing the ATC codes, drug type and drug name of the medication registered for HZ and PHN. (DOCX 14 kb) [file 12879_2018_3121_MOESM1_ESM.docx]

Additional file 1.

Word format, File extension .doc

Additional table 1. Medication registered for HZ and PHN (ATC codes).

Table shows the ATC codes, drug type and drug name of the medication registered for HZ and PHN.

| **ATC code** | **Drug type** | **Drug name** |
| --- | --- | --- |
| J05A | Direct acting antivirals | Aciclovir |
|  |  | Famciclovir |
|  |  | Valaciclovir |
| N02A | Opioids | Tramadol |
| N03 | Antiepileptics | Phenytoin |
|  |  | Carbamazepine |
|  |  | Gabapentin |
|  |  | Pregabalin |
| N06A | Antidepressant | Amitriptyline |
|  |  | Nortriptyline |
|  |  | Imipramine |
|  |  | Desipramine |
| N01B | Local Anesthetics | Capsaicin |
|  |  | Lidocaine |
