# Supplementary material for: Risk and impact of herpes zoster among COPD patients: a population-based study, 2009–2014
Source: BMC Infect Dis. 2018 May 3;18:203. doi: 10.1186/s12879-018-3121-x (PMC5934818; doi:10.1186/s12879-018-3121-x)
Supplement: Supplementary file 2 — Generalized linear models. Table including the different statistical GLM utilized to compare the COPD and the non-COPD populations depending on the outcome variable. (DOCX 15 kb) [file 12879_2018_3121_MOESM2_ESM.docx]

Additional file 2.

Word format, File extension .doc

**Additional table 2.** Generalized linear models.

Table shows the different statistical GLM utilized to compare the COPD and the non-COPD populations depending on the outcome variable.

| **Outcome** | **Regression** | **Adjustment** |
| --- | --- | --- |
| **Table 4** |  |  |
| Number of outpatient visits for HZ | Negative Binomial^a^ | COPD (No COPD/COPD/COPD-ICS), Gender, Year, Age and Health Department |
| Number of drug prescriptions for HZ | Negative Binomial^a^ | COPD (No COPD/COPD/COPD-ICS), Gender, Year, Age and Health Department |
| HZ hospitalizations (0: Non Hospitalized; 1: At least one HZ hospitalization) | Logistic | COPD (No COPD/COPD/COPD-ICS), Gender, Year, Age and Health Department |
| Logarithm^b^ hospital stay length (days) for HZ | Lineal | COPD (No COPD/COPD/COPD-ICS), Age and Health Department |
| Logarithm^b^ of sick leave (days) for HZ | Lineal | COPD (No COPD/COPD/COPD-ICS) and Age |
|  |  |  |
| **Table 5** |  |  |
| Number of COPD outpatient visits | Negative Binomial^a^ | Period (Pre/Post), Gender, Year, Age and Health Department |
| COPD Hospitalizations (0: Non Hospitalized; 1: At least one HZ hospitalization) | Logistic | Period (Pre/Post), Gender and Age |
| Logarithm^b^ of hospital stay length (days) for COPD | Lineal | Period (Pre/Post), Year, and Health Department |
| Number of ICS prescriptions | Negative Binomial^a^ | Period (Pre/Post), Gender, Year, Age and Health Department |

^a^ Negative binomial distributions were considered to solve the overdispersion problem.

^b^ Lognormal distributions were assumed because our data were highly right skewed.
